# Fecal Deployment: An Alternative Way of Defensive Host Plant Cardenolide Use by *Lilioceris merdigera* Larvae

**DOI:** 10.1007/s10886-023-01465-8

**Published:** 2023-12-07

**Authors:** Michael Baum, Susanne Dobler

**Affiliations:** 1 Molecular Evolutionary Biology, Institute of Zoology, Universität Hamburg, Martin-Luther-King-Platz 3, 20146 Hamburg, Germany; 2 Chemistry Education Department, IPN, Leibniz Institute for Science and Mathematics Education, Olshausenstraße 62, 24118 Kiel, Germany

**Keywords:** Insect-plant interaction, Host plant cardenolide, Fecal shield, Insect defense, Leaf beetle

## Abstract

The brilliant red *Lilioceris merdigera* (Coleoptera, Chrysomelidae) can spend its entire life cycle on the cardenolide-containing plant *Convallaria majalis* (lily of the valley) and forms stable populations on this host. Yet, in contrast to many other insects on cardenolide-containing plants *L. merdigera* does not sequester these plant toxins in the body but rather both adult beetles and larvae eliminate ingested cardenolides with the feces. Tracer feeding experiments showed that this holds true for radioactively labeled ouabain and digoxin, a highly polar and a rather apolar cardenolide. Both compounds or their derivatives are incorporated in the fecal shields of the larvae. The apolar digoxin, but not the polar ouabain, showed a deterrent effect on the generalist predatory ant *Myrmica rubra*, which occurs in the habitat of *L. merdigera*. The deterrent effect was detected for digoxin both in choice and feeding time assays. In a predator choice assay, a fecal shield derived from a diet of cardenolide-containing *C. majalis* offered *L. merdigera* larvae better protection from *M. rubra* than one derived from non-cardenolide *Allium schoenoprasum* (chives) or no fecal shield at all. Thus, we here present data suggesting a new way how insects may gain protection by feeding on cardenolide-containing plants.

**Supplementary Information:**

The online version contains supplementary material available at 10.1007/s10886-023-01465-8.

## Introduction

The co-evolutionary arms race between plants developing defense mechanisms (Mithöfer et al. 2018) and herbivorous insects overcoming them (Caprio and Tabashnik 1992; War et al. 2020) has led to many intriguing adaptations in the ways how insects deal with potentially toxic plant compounds. Herbivorous insects from different orders utilize the plant’s chemical defense as protection against predators (Duffey 1980; Scudder et al. 1986; Opitz and Müller 2009). Cardenolides, an extensively studied group of specialized plant metabolites found in at least 12 different angiosperm plant families (Agrawal et al. 2012), are sequestered for defensive purposes by numerous insect species (Euw et al. 1967; Cohen and Brower 1983; Malcolm 1990; Dobler et al. 1998). In a number of these cardenolide-sequestering species, convergent amino acid substitutions evolved in the Na,K-ATPase, a ubiquitous and essential ion pump, rendering this main target site of cardenolides insensitive to the toxins (Dobler et al. 2012, 2019; Zhen et al. 2012; Karageorgi et al. 2019; Taverner et al. 2019; Yang et al. 2019). Another possible way of coping with a cardenolide-containing diet are epithelial barriers (Dobler et al. 2015) which can exclude toxins from susceptible tissues (Petschenka et al. 2013; Dermauw and Van Leeuwen 2014). The effectivity of such barriers strongly depends on the cardenolide’s polarity influencing its ability to cross lipid cell membranes, and the presence and type of transmembrane carrier proteins actively transporting cardenolides (Groen et al. 2017; Kowalski et al. 2020).

The Onion Leaf Beetle *Lilioceris merdigera* (Coleoptera, Chrysomelidae) feeds on several different plant species of the genera *Allium*, *Convallaria*, *Lilium* and *Polygonatum* (Haye and Kenis 2004), among them the cardenolide-rich *C. majalis*. *C. majalis* is known to contain a mixture of at least 38 different cardenolides derived from nine aglycones with the most abundant of them being convallatoxin (Kopp and Kubelka 1982). The beetle’s strategy to circumvent the toxic effects is yet unknown, as well as the fate of the cardenolides ingested by the animals. In this study, we investigated how *L. merdigera* deals with ingested cardenolides in larval and adult stages. Adult *L. merdigera* produce defensive secretions in glands situated on their pronotum and elytra, as is the case for many other chrysomelids (Deroe and Pasteels 1982) while, like other species within the Chrysomelidae, *L. merdigera* larvae carry fecal matter on their dorsal surface (Walsh and Riley 1868; Eisner et al. 1967; Olmstead 1994; Chaboo et al. 2007).We performed tracer feeding studies with both adults and larvae using ³H-labeled ouabain and digoxin, two cardenolides that differ in polarity. Tracer feeding results show an almost complete excretion of ingested cardenolides via the beetles’ feces. Bioassays with the co-occurring predatory ant *Myrmica rubra* were performed to test if *L. merdigera* larvae profit from feeding on cardenolide-containing plants by an increased deterrent effect of their fecal shields towards predators. Further assays investigated deterrent effects of the two cardenolides on the predatory *M. rubra*.

## Methods and Materials

### Collection and Rearing of Animals

Individuals of *L. merdigera* were collected as larvae or adults near Duvenstedt, Hamburg (53.71° N, 10.09° E) on *Convallaria* and near Klein Schmölen (53.13° N, 11.30° E) on *Allium* in May and June. Only every second spotted individual was taken to avoid affecting the population. They were split in two groups and kept in terraria in the laboratory on each of the two plants. Terraria were filled with 4 cm wet vermiculite to help sustain a constant level of humidity. *C. majalis* was collected at University Hamburg (53.57° N, 9.97° E); *Allium schoenoprasum* (chive) was bought in “organic” quality presumably free of artificial pesticides. Beetles were kept at 20 °C and a 16 L:8D photoperiodic regime in a climate chamber. Freshly laid eggs were collected and transferred to fresh leaves in small petri dishes. Hatched larvae were again transferred to separated containers with fresh plant leaves and vermiculite until used in experiments.

The ant *Myrmica rubra* was chosen as a potential predator of *L. merdigera* larvae due to its opportunistic prey spectrum (Radchenko and Elmes 2010), which includes all kinds of insect larvae, and its general abundance in many habitats (Wetterer and Radchenko 2011), including at least one of the abovementioned collection sites of *L. merdigera* (Baum 2016). Three ant colonies were purchased from the web shop antstore.net (Berlin, Germany) and kept in a formicarium with three identical units at 20 °C under a 16 L:8D photoperiodic regime. Each unit had a polyethylene arena where food (honey and dead insects) and water were provided. The arena floors were covered with sand. Ants were kept from escaping by a paraffin oil border, applied with a brush on the arena’s walls and lid. A 1 cm diameter hose connected the arena with the nest, which was carved into an aerated concrete block.

### Tracer Feeding

Experiments were conducted using ^3^H-ouabain and ^3^H-digoxin (PerkinElmer). To track the fate of ingested cardenolides, pieces of *Convallaria* leaves (~ 2 cm^2^) were coated with 5 µl ethanolic solution of the respective ^3^H-labeled cardenolide (stock solution diluted 1:20 and spiked with 10 mM unlabeled cardenolide) and offered to a second to third instar larva (n = 3 and 7 respectively) or an imago (n = 7 and 6 respectively) in a sealed petri dish. After 4 days or complete consumption of the leaf, the animal was offered a piece of non-labeled leaf and the labeled one was removed. After feeding two more days on the non-labeled leaf, the following samples were collected: (1) All unconsumed leaf parts including the labeled ones, (2) the imago or larva without fecal shield, (3) all feces collected in the dish including the fecal shield, and (4) 100 µl MeOH used to rinse the dish after all other samples were collected. The samples, except the fourth one, were frozen on liquid N_2_, ground to powder and dissolved in 200 µl MeOH. After thorough vortexing, the samples were sonicated in an ultrasonic bath (Sonorex RK102, Bandelin) for 5 min for increased extraction. After a short spin in a centrifuge (< 5,000 x g), the supernatants and the rinse sample (4) were transferred to scintillation vials. 3 ml of scintillation cocktail (Ultima Gold XR, PerkinElmer, Waltham, USA) were added and the samples vortexed. The amount of ^3^H in each sample was determined on a liquid scintillation counter (Wallac 1409, PerkinElmer). The samples of leaves and rinse served as control for the recovery of the total applied amount of ^3^H. The combined amount of ^3^H recovered from the beetle and its feces was regarded as having been ingested by the insect during the experiment. The proportions of dpm_beetle_/dpm_beetle+feces_ and dpm_feces_/dpm_beetle+feces_ were compared using a paired-sample *t-test*. Data from one treatment was not normally distributed and a non-parametric *Wilcoxon signed-rank test* was applied.

### Bioassays with Ants

Three ant colonies were used in feeding experiments. To minimize potential effects of learning or preferences of one colony, the order of runs was randomized using the RAND() function of MS Excel(1), each assay run was randomly assigned to one of the three colonies using the same function(2). At the beginning of each run, positioning of the sample in the arena was randomized as follows (3): a foraging ant in the arena was arbitrarily selected and followed for 30 s. The dish was then put at the position where the ant was located after 30 s of foraging.

To determine effects of cardenolides on *M. rubra* feeding, three different bioassays were performed: a honey choice assay, a feeding time assay and a predator choice assay. Prior to the assays, all available food sources were removed from the *M. rubra* colonies’ arenas. In the first two assays, ants were offered honey with and without cardenolides in two wells on the same clay dish (Online Resource 1a). Ouabain and digoxin were used, as they represent hydrophilic and rather hydrophobic cardenolides. As digoxin had to be dissolved in DMSO, an equivalent amount of the solvent was also added to the cardenolide-free honey on the digoxin dishes. Thirty minutes after positioning of the dish, the number of ants feeding on each well were counted and regarded as a measure of attractiveness of the well for the colony. The assay was performed with different concentrations (1 mM and 5 mM) and with 11 to 15 replicates for each concentration of each cardenolide. Statistical analysis was performed on pooled data from all three colonies. As the data were not normally distributed, a non-parametric one-tailed *Wilcoxon signed-rank test* with continuity correction for paired data was applied.

A feeding time assay was used to investigate possible effects of cardenolides on individual ants. After positioning the dish with honey and 1mM cardenolide honey in the arena, it was filmed for 30 min. Afterwards the films were analyzed and feeding duration of individual *M. rubra* workers were recorded. Feeding duration was defined as the time between an ant lowering its head into the honey well and the ant walking away from the well. In six 30 min runs, the feeding duration of a total number of 130 ants (ouabain) and 132 ants (digoxin) were recorded. A two-tailed *Wilcoxon rank-sum test* with continuity correction was performed.

The impact of the cardenolide-loaded fecal shield of *L. merdigera* larvae on *M. rubra* was tested in a predator choice assay by offering the ants four differently treated late instar larvae on a small petri dish (see Supplementary Material 3): two larvae were reared on *Allium* and were therefore regarded devoid of cardenolides; two were reared on cardenolide-containing *Convallaria* before they were killed by freezing. One larva of each group had its fecal shield removed. The first larva carried away from the dish by ants was considered “chosen” and all larvae were removed from the arena. If no larva was chosen after 30 min, the dish was removed and all larvae considered “not chosen”. After a total of 67 runs, the contingency table of the pooled data from three ant colonies was analyzed for independence of treatment using *Fisher’s exact test*. The fact that cardenolides were shown to be potentially deterrent in the honey choice experiments allowed for one-sided statistical tests, ignoring potentially attractant effects of the fecal defense. Treatments were compared with one-tailed pairwise *Fisher’s Exact tests*.

## Results

### Tracer Feeding

**Fig. 1:**
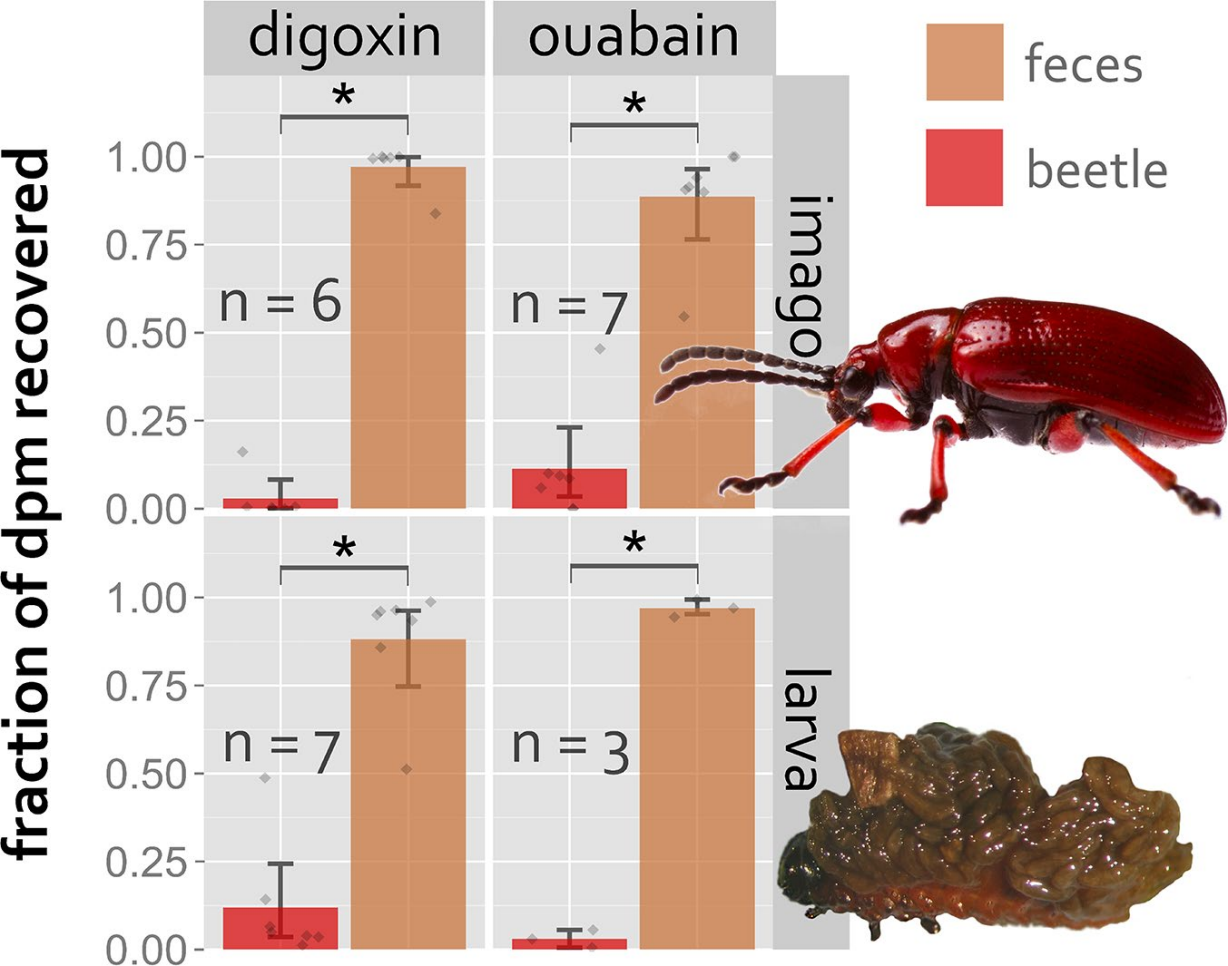
Recovery of orally administered ³H-labeled cardenolides from feces or bodies of ***L. merdigera*** imagines and larvae; animals were fed unlabeled leaves for 2 days prior to extraction; * marks P < 0.05; (Picture of imago courtesy of Samuel Waldron)

The radioactivity ingested by imagines and larvae of *L. merdigera* was to a large extent recovered from the beetles’ feces (Fig. 1, additional data in Supplementary Material 1). In two of the 23 beetles, one ouabain-fed imago and one digoxin-fed larva, about half of the radioactivity was recovered from their body. In all treatment conditions, tests showed significantly more ³H recovered from the feces than from the beetles (P < 0.05).

### Bioassays

The repellent effect of the two cardenolides on a generalistic predatory arthropod was tested in two assays with *M. rubra*. When being offered diluted honey with and without ouabain or digoxin, after 30 min more ants were found on average feeding on the control solution than on the cardenolide solution in every setup. The difference was statistically significant (P < 0.005) for both concentrations of digoxin, however the differences between honey and cardenolide honey were not statistically significant (P > 0.05) for both ouabain concentrations (Table 1).

**Table 1:** Food choice assay with *Myrmica rubra* and honey water enriched with cardenolides or just the solvent; feeding ants were counted at each well (control/cardenolide) 30 min after honey was offered

cardenolide	c_cardenolide_ [mM]	N_ants_ ± SE (control)	N_ants_ ± SE (cardenolide)	n	P
ouabain	1	2.85 ± 0.18	2.77 ± 0.14	13	0.543
	5	2.36 ± 0.25	1.55 ± 0.14	11	0.117
digoxin	1	2.33 ± 0.16	1.07 ± 0.13	15	0.002**
	5	2.00 ± 0.21	0.42 ± 0.16	12	0.004**

**marks P < 0.005

**Fig. 2:**
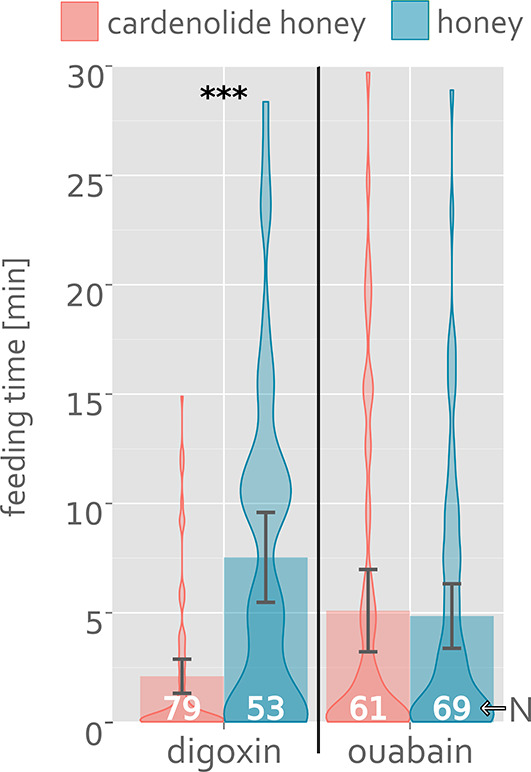
Feeding time assay with ***M. rubra*** and honey water enriched with 1mM cardenolides (red) or just the solvent (blue) in a simultaneous choice test; bars represent means with bootstrapped 95% confidence intervals; violin plots show frequency distributions of observed feeding times; N is number of ants; *** marks P < 0.001

In a second honey feeding assay, the time individual ants spend feeding on diluted honey with and without 1mM ouabain or digoxin was measured. Ants were frequently observed cleaning their antennae after contact with digoxin. Significant differences in feeding time between cardenolide and control honey were found when digoxin was offered, but not when ouabain was offered (Fig. 2).

**Fig. 3:**
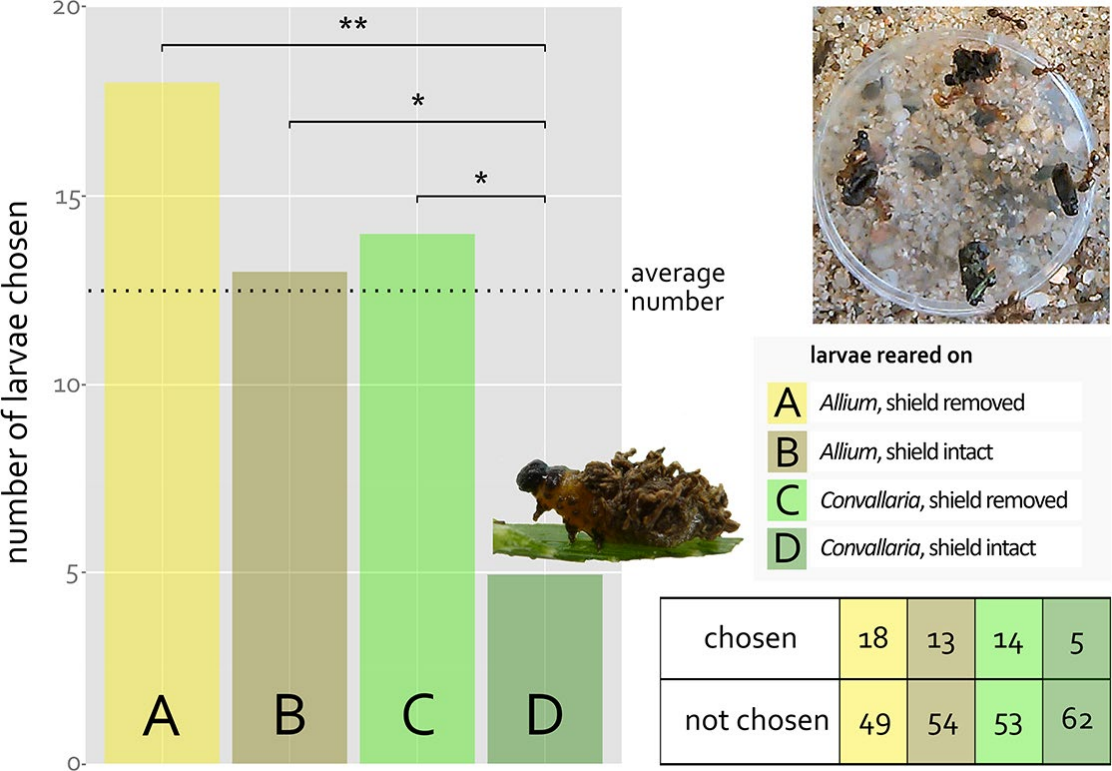
Predator choice assay with ***M. rubra*** and ***L. merdigera*** larvae; four larvae reared on ***A. schoenoprasum*** with fecal shield removed (**A**) or intact (**B**) and reared on ***C. majalis*** with fecal shield removed (**C**) or intact (**D**) were offered to ***M. rubra*** colonies; first larvae carried away by the ants was considered “chosen”; * and ** mark P < 0.05 and P < 0.005 respectively

In a predator choice assay, ants were offered beetle larvae reared on *Allium* or *Convallaria.* Independence of the predators’ choice from treatment of the larvae was rejected. Larvae reared on *A. schoenoprasum* and larvae without fecal shield were chosen significantly more often than larvae with an intact *C. majalis*-derived fecal shield (Fig. 3).

## Discussion

The brilliant red *L. merdigera* can spend its entire life cycle on the cardenolide-containing plant *C. majalis* and forms stable populations on this host. Yet, it does not have any of the well-known resistance-conferring substitutions in its Na,K-ATPase that repeatedly evolved in other chrysomelid species (Agrawal et al. in prep.). As we show here, *L. merdigera* does not sequester plant-derived cardenolides in its body but rather both adult beetles and larvae eliminate ingested cardenolides with the feces. This holds true for ouabain and digoxin, a highly polar and a rather apolar cardenolide. Adult *L. merdigera* therefore do not seem to sequester host plant cardenolides into their defensive secretory glands, as other chrysomelids do (Dobler et al. 1998). In addition, the secretions of several chrysomeline leaf beetle species contain endogenously produced cardenolides, while those of the criocerine *Lilioceris lilii* contains phenylalanine derivatives. (Pasteels et al. 1989). Similar amino acid-derived defensive compounds are assumed to occur in the secretions of adult *L. merdigera*. Yet, both tested cardenolides or their derivatives are incorporated in the fecal shield of the larvae.

Fecal shields in chrysomelid larvae are known from several groups (Cassidinae, Clytrinae, Criocerinae, Cryptocephalinae) and have repeatedly shown to contain plant derived compounds (Olmstead and Denno 1992; Morton and Vencl 1998; Gómez et al. 1999; Keefover-Ring 2013). As sequestration has been defined as “the selective uptake, transport, modification, storage and deployment of plant secondary chemicals for the insect’s own defense” (Heckel 2018), a recycling of plant compounds in fecal shields cannot per se be called sequestration as long as no modification or selectivity has been shown. Our tracer feeding studies show that ingested cardenolides or their metabolized derivatives are in large part found in the feces of larval and adult *L. merdigera*. The radioactivity recovered from the body of a small number of individual beetles might represent remnants of frass not yet discarded from the gut. During gut transit, ingested cardenolides can undergo metabolisation leading to altered chemical properties. Such metabolic reactions are described for cardenolides in the guts of adapted butterflies (Marty and Krieger 1984; Abe et al. 1996) and the accumulation of compounds in the midgut is generally suspected to be a necessary precondition for sequestration (Petschenka and Agrawal 2015). While we do not know whether the radioactively labeled ouabain and digoxin were metabolically altered during the gut passage or if an active accumulation in the midgut takes place, our predator choice assays support that ingested cardenolides or their derivatives are excreted in deterrent forms in the feces of *L. merdigera*.

Of the two cardenolides tested on the predatory ant *M. rubra*, the ants did not discriminate significantly between honey plus ouabain and pure honey. Digoxin, on the other hand, clearly had a deterrent effect on *M. rubra*. Ants were frequently observed cleaning their mandibles and antennae after contact with digoxin, a reaction well-known to be caused in ants by chemical deterrents (Eisner and Meinwald 1966; Nogueira-de-Sá and Trigo 2005). The difference in the effect of both cardenolides is supposedly determined by their chemical properties, namely their polarity and thus their differing ability to penetrate cell membranes and epithelia. This may have an influence on their propensity to get in contact with ants’ receptor proteins. Though insect odor receptors generally detect hydrophobic substances (Leal 2013), gustatory receptors are also known to detect hydrophilic compounds (Freeman et al. 2014). Malcolm (1991) reports that ouabain can be tasted by humans only at concentrations 40 times higher than the less polar cardenolide digitoxin. The polarity differences are also believed to account for the different toxic effects of orally ingested cardenolides on cats, where ouabain only showed emetic effects at a dose six times higher than the lethal dose of digoxin (Malcolm 1991).

When confronted with larvae of *L. merdigera* wearing plant-derived fecal shields or having their fecal shield removed, *M. rubra* chose the unshielded and *Allium*-reared shielded larvae considerably more often than the ones with cardenolide-containing *Convallaria*-derived fecal shields. Future experiments may consider possible confounding factors of the ants’ choice like size of the larva or their fecal shield. Chemical protection by fecal defenses was repeatedly shown to be based on host-derived compounds (Morton and Vencl 1998; Vencl et al. 2005) and *M. rubra* was also deterred by those in *Eurypedus*’ defense derived from *Cordia curassavica* (Gómez et al. 1999). None of the reports known to us includes host plant cardenolides or their metabolites as deterrent fecal compounds.

Fecal shields of chrysomelid larvae were, in other instances, reported to attract predatory and parasitic insects rather than deterring them (Schaffner and Müller 2001; Müller and Hilker 2004). In our assays five larvae with *Convallaria*-derived fecal shield were chosen by the ants, which may be explained by the fluctuating cardenolide content of *C. majalis* leaves (Schrutka-Rechtenstamm et al. 1985), potentially leading to diminished deterrent effects in individual fecal shields. Adding the findings of the honey choice experiments, we suggest that rather apolar cardenolides from *C. majalis* or their derivatives in the fecal shield of *L. merdigera* larvae reduce predation by *M. rubra*.

Surprisingly, no statistical difference in predator choice was discovered between *Allium*-reared larvae with and without fecal shield (18:13 chosen larvae). Compounds of *Allium* species are reported to effectively repel insects like the pest beetle *Callosobruchus maculatus* (Denloye 2010), *Anopheles* mosquitoes (Denloye et al. 2003) or camel botflies (Khater 2014). The effective chemical protection of larvae of the cassidine beetle *Chelymorpha reimoseri* is reported to be independent of their fecal defense (Bottcher et al. 2009). However, this seems not to be the case in *L. merdigera*, as the *Allium*-fed larvae were regularly chosen by the ants irrespective of having a fecal shield, thus not providing evidence of significant protection against *M. rubra* by *Allium*-derived fecal shields. Whether potentially deterrent *Allium* metabolites are at all present in the feces or have possibly been detoxified in the beetles’ guts remains to be investigated. As the efficacy of fecal protection can strongly depend on the predator species (Müller and Hilker 2004), protection towards other predators and visual protection by using feces as camouflage (Jones 1994; Müller and Hilker 2004) cannot be ruled out for *Allium*-derived fecal shields. Our results underline the strong dependence of fecal coverages’ defensive properties on the specific host plant and add another instance to the already known versatile forms of cardenolide usage by insect herbivores.

The physiological adaptations allowing *L. merdigera* to use cardenolides or their derivatives in the fecal shield need future investigations. The fact that the ingested radioactive tracers were almost completely found in feces points at an effective barrier in the beetle’s gut. Retention of compounds in the gut can be accomplished by preventing them from crossing the gut wall in the first place, by efficient excretion from the hemolymph via Malpighian tubules, or by a combination of both processes. Barbehenn (2001) proposes micellar complexes formed by the apolar cardenolide digitoxin and phospholipids at the peritrophic membrane as a mechanism to decrease cardenolide permeability of the insect gut and expects “an efficient detoxification [mechanism] in the midgut epithelium”. Such a mechanism is likely to include members of the ABCB transport protein subfamily. There is evidence of these proteins being involved in compartmentalization of cardenolides in different species (Petschenka et al. 2013; Dobler et al. 2015; Groen et al. 2017; Kowalski et al. 2020). In addition, convallatoxin, the most abundant cardenolide in *C. majalis* (Kopp and Kubelka 1982), has been shown to be a substrate of an ABCB transport protein in rats (Gozalpour et al. 2014).

The use of host plant compounds for defensive purposes can be regarded as shift of a chemical defense strategy to a higher trophic level (Petschenka and Agrawal 2015) at certain costs, representing a seesaw between predator pressure and physiological effects and costs (Züst et al. 2018; Carlson and Agrawal 2023). In the case of fecal defense, the protective advantages are linked to disadvantages like possible attraction of parasites and certain predators (Müller and Hilker 2004) such that fecal defense was described as a “double-edged sword” (Huang et al. 2023). Due to its ability to live on plants with strongly differing specialized metabolites, *L. merdigera* may pose a future model to better investigate this “evolutionary dilemma” (Müller and Hilker 2000) in combination with host plant preferences.

### Electronic Supplementary Material

Below is the link to the electronic supplementary material.

Supplementary Material 1

Supplementary Material 2

Supplementary Material 3

## Declarations

This work was supported by Deutsche Forschungsgemeinschaft (DFG) (Do527/5 − 2) and the John Templeton Foundation (#41855).
