# Supplementary material for: Fecal Deployment: An Alternative Way of Defensive Host Plant Cardenolide Use by Lilioceris merdigera Larvae
Source: J Chem Ecol. 2023 Dec 7;50(1-2):63–70. doi: 10.1007/s10886-023-01465-8 (PMC10991028; doi:10.1007/s10886-023-01465-8)
Supplement: Supplementary file 2 — Supplementary Material 2 [file 10886_2023_1465_MOESM2_ESM.docx]

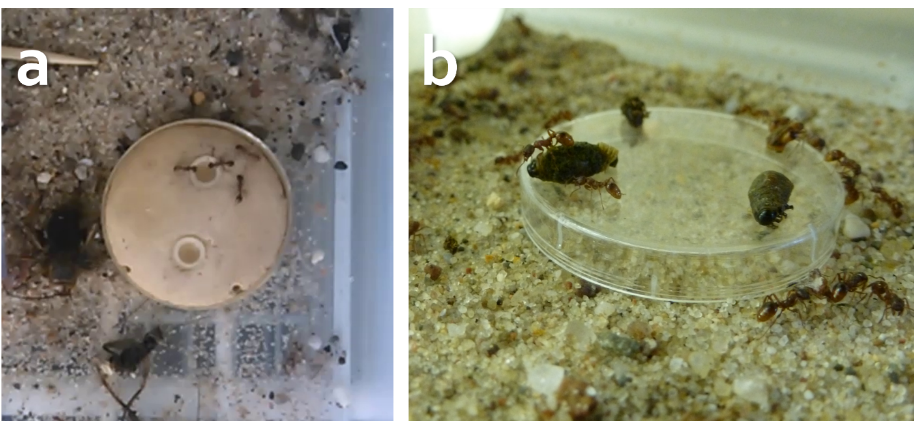
SUPPLEMENTAry

Online Resource 1a Setup of cardenolide honey choice assay with *M. rubra*; both wells contained honey, one of them enriched with cardenolides; b predator choice assay: ants were offered four dead *L. merdigera* larvae of which two had fed on *Allium* and two had fed on *Convallaria*, with one larva of each group possessing an intact fecal shield and the other having it removed (SM1.tif)

**Online Resource 2** Supplementary data of the tracer feeding experiment including non-ingested tritium radiation recovered from leaves and dishes (SM2.xlsx)
