# Supplementary figures and images for: Fecal Deployment: An Alternative Way of Defensive Host Plant Cardenolide Use by Lilioceris merdigera Larvae
Source: J Chem Ecol. 2023 Dec 7;50(1-2):63–70. doi: 10.1007/s10886-023-01465-8 (PMC10991028; doi:10.1007/s10886-023-01465-8)

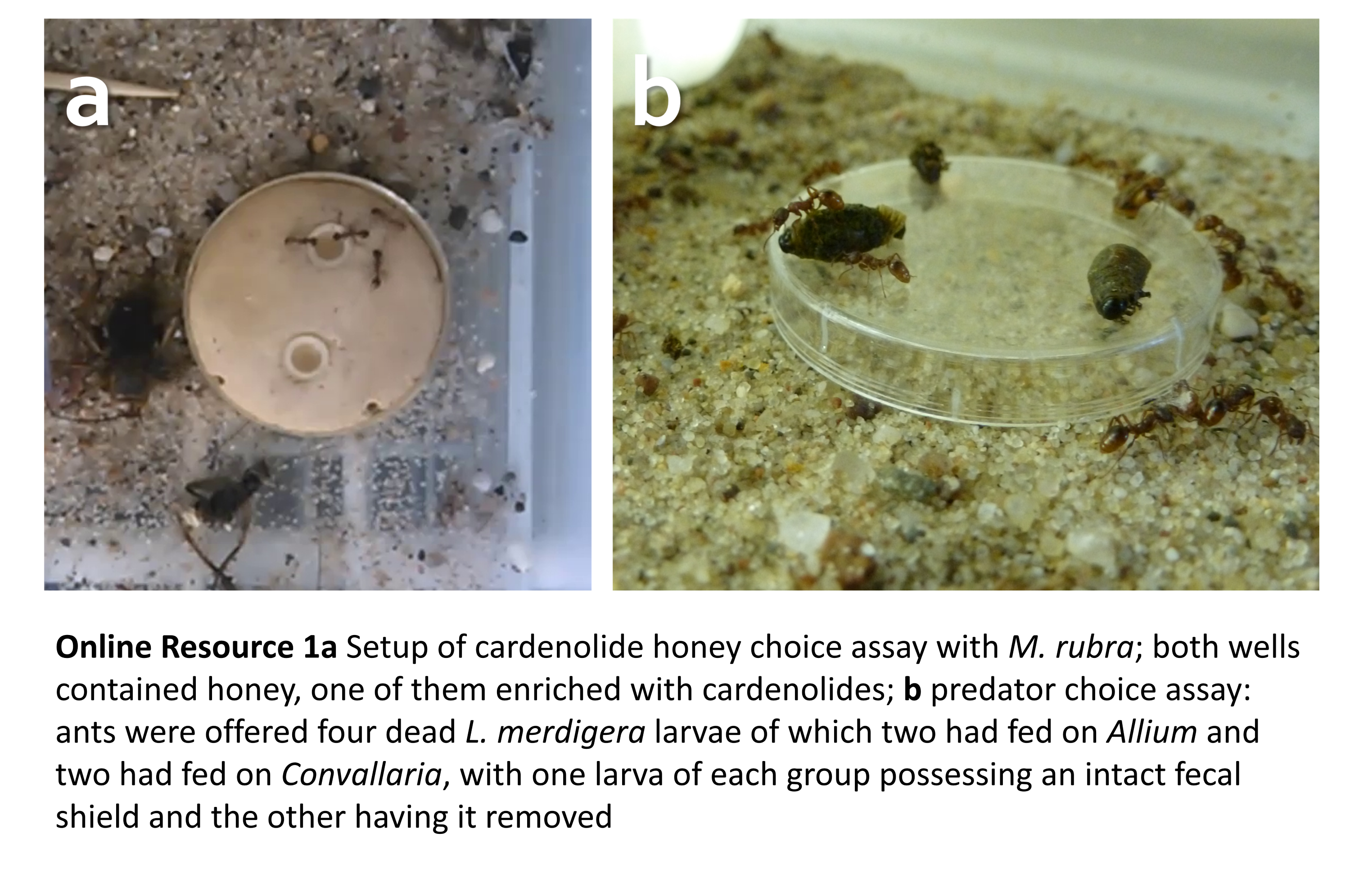

Supplement: Supplementary file 3 — Supplementary Material 3 [file 10886_2023_1465_MOESM3_ESM.tif]
